# Recent Developments in Graphene-Based Toxic Gas Sensors: A Theoretical Overview

**DOI:** 10.3390/s21061992

**Published:** 2021-03-11

**Authors:** Heriberto Cruz-Martínez, Hugo Rojas-Chávez, Fernando Montejo-Alvaro, Yesica A. Peña-Castañeda, Pastor T. Matadamas-Ortiz, Dora I. Medina

**Affiliations:** 1 Tecnológico Nacional de México, Instituto Tecnológico del Valle de Etla, Abasolo S/N, Barrio del Agua Buena, Santiago Suchilquitongo, Oaxaca 68230, Mexico; heri1234@hotmail.com (H.C.-M.); moaf1217@gmail.com (F.M.-A.); 2 Tecnológico Nacional de México, Instituto Tecnológico de Tláhuac II, Camino Real 625, Tláhuac, Ciudad de México 13508, Mexico; rojas_hugo@ittlahuac2.edu.mx; 3 Colegio de Ciencia y Tecnología, Universidad Autónoma de la Ciudad de México, Av. Fray Servando Teresa de Mier 92, Cuauhtémoc, Ciudad de México 06080, Mexico; yesica.pena@uacm.edu.mx; 4 Instituto Politécnico Nacional, CIIDIR-OAXACA, Hornos No. 1003, Noche Buena, Santa Cruz Xoxocotlán 71230, Mexico; 5 Tecnologico de Monterrey, School of Engineering and Sciences, Atizapan de Zaragoza, Estado de México 52926, Mexico

**Keywords:** pristine graphene, defective graphene, doped graphene, density functional theory, first principle studies, toxic gas sensors, adsorption energy

## Abstract

Detecting and monitoring air-polluting gases such as carbon monoxide (CO), nitrogen oxides (NO_x_), and sulfur oxides (SO_x_) are critical, as these gases are toxic and harm the ecosystem and the human health. Therefore, it is necessary to design high-performance gas sensors for toxic gas detection. In this sense, graphene-based materials are promising for use as toxic gas sensors. In addition to experimental investigations, first-principle methods have enabled graphene-based sensor design to progress by leaps and bounds. This review presents a detailed analysis of graphene-based toxic gas sensors by using first-principle methods. The modifications made to graphene, such as decorated, defective, and doped to improve the detection of NO_x_, SO_x_, and CO toxic gases are revised and analyzed. In general, graphene decorated with transition metals, defective graphene, and doped graphene have a higher sensibility toward the toxic gases than pristine graphene. This review shows the relevance of using first-principle studies for the design of novel and efficient toxic gas sensors. The theoretical results obtained to date can greatly help experimental groups to design novel and efficient graphene-based toxic gas sensors.

## 1. Introduction

The conversion of energy from one form to another many times affects the air composition in several ways. It is well-known that fossil fuels have been powering industrial development and the amenities of modern life that we enjoy. However, the combustion of fossil fuels contributes to a great extent to composition variations of the atmosphere, and this is mainly due to harmful gas emissions.

Harmful gases include, for instance, aliphatic hydrocarbons, carbon monoxide (CO), nitrogen oxides (NO_x_), and sulfur oxides (SO_x_), among others. In this context, health expenditures have increased due to air pollution, which is mainly associated with the rapid industrialization of many countries. Consequently, the disruption of ecological balance and serious public health issues caused by harmful gases are raising global concerns [1,2]. Table 1 illustrates some aspects about the harmful effects of toxic gases related to human health.

On the other hand, until today, a sizable number of countries depend on oil for energy uses and development. Consequently, harmful effects on the environment such as global warming, ozone depletion, acid rain and climate change may result from the gases emanating from fossil fuel combustion. Therefore, harmful gases do not only affect human health, but they also have an undesirable impact on the environment.

**Figure 1:**
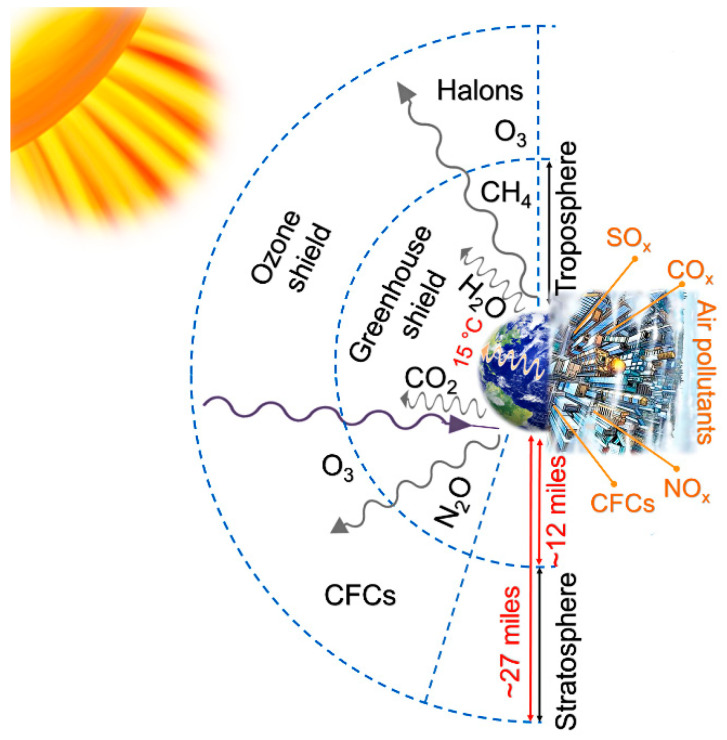
Model of the Earth′s gaseous protective shield [12,16].

According to Springer, the greenhouse effect of the troposphere is beneficial because it makes the earth habitable at an overall average temperature of about 15 °C [12] (see Figure 1). However, higher concentrations of CO_2_, methane, water vapor, chlorofluorocarbons (CFCs), ozone, and nitrous oxide in the upper atmosphere could result in global warming, accompanied by economic and environmental implications [12,13,14,15,16].

Against this backdrop, effectively sensing and capturing these harmful gases, such as CO, NO_x_, and SO_x_, can greatly help protect the environment and human health [9,17]. Nowadays many materials, such as metal oxide semiconductors, conducting polymers, carbon-based materials, have been investigated and utilized as toxic gas sensors [18,19,20]. However, the challenges of these gas sensors can be one or more of the following: cost, sensitivity (e.g., ppb level is rare), and poor selectivity, among others [19]. Therefore, it is necessary to design high-performance gas sensors for detecting these toxic gases. As an alternative, among the carbon materials, graphene, a 2D monolayer form of sp^2^-hybridized carbon atoms, could prove a key material for sensing applications due to its exceptional thermal conductivity, high electron mobility, excellent mechanical properties, and high specific surface area [21,22,23,24]. Due to its remarkable properties, graphene opens up a wide range of promising applications in the sensors field, from fundamental science to industrial applications [25,26,27,28,29]. However, the main issue is that gas molecules are weakly adsorbed on graphene due to its low reactivity [30,31,32]. For this reason, both at the theoretical and experimental levels, several strategies have been developed to modify the electronic and structural properties of graphene and, consequently, improve its reactivity toward the toxic gases. Such strategies include doped, decorated, defective, and functionalized graphene [33,34,35].

Experimentally, the current conception of novel graphene sensor materials and the required performance improvements are largely limited by lack of rapid and economical synthesis routes and post-testing strategies to ensure their functionality. Currently, different synthesis techniques such as chemical vapor deposition, sputtering, drop casting, spin coating, and inkjet printing have been used to fabricate high quality graphene for the detection of toxic gases [36,37]. However, many of these methods are expensive and not easily scalable for mass production. In addition, it is difficult to control the doping concentration and the number of graphene layers. Undoubtedly, such limitations can be overcome if sensor materials are designed, modeled, and evaluated from a theoretical point of view (e.g., first-principle methods). The first-principle or ab initio methods are based on the quantum mechanics theory. Specifically, the density functional theory (DFT) is primarily a formalism of electronic ground state structure, couched in terms of the electronic density distribution [38]. The DFT-based simulations are essential for explaining and understanding the experimental results at the molecular level, or as a predictive tool for the rational design of novel gas sensors [39]. The DFT calculations provide important information, such as the adsorption mechanisms, the adsorption energy, charge transfer, electronic modification after gas adsorption, feasible approaches to enhance adsorption or desorption, that are critical for designing novel gas sensors [40]. Due to the critical role that theoretical calculations have in the design of toxic gas sensors, numerous DFT studies have been conducted to investigate novel graphene-based gas sensors. However, to date there are no detailed and critical reviews of the current progress in theoretical design of graphene-based toxic gas sensors; state-of-the-art reviews mainly focus on experimental evidence [28,29,41,42]. Therefore, this review presents a detailed and critical analysis of the progress of graphene-based toxic gas sensors by using first-principle methods. The modifications made to graphene, such as defective, doped, and decorated to improve the detection of CO and NO_x_, and SO_x_ toxic gases, are revised and analyzed in detail.

## 2. Pristine Graphene

**Figure 2:**
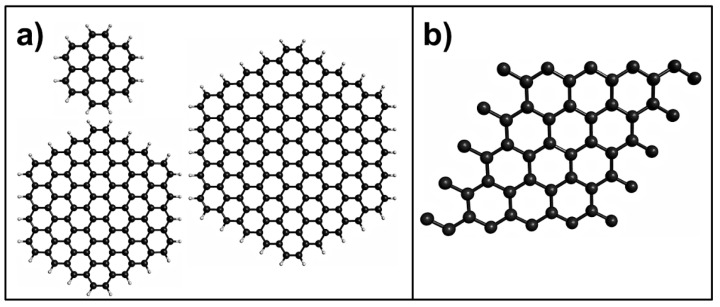
Models of graphene used for the density functional theory (DFT) calculations: (**a**) aromatic molecules, (**b**) graphene supercell. Black and gray spheres represent C and H atoms, respectively.

Different approaches have been used for theoretical studies into pristine graphene, such as aromatic molecules (finite system) and periodic systems (supercell) (see Figure 2). There have been several theoretical studies conducted on the use of pristine graphene as a toxic gas sensor [43,44,45,46]. One of the first DFT-based studies on the use of pristine graphene as a toxic gas sensor was performed by Leenaerts et al. [43]. They investigated the adsorption of CO, NO_2_, and NO on pristine graphene using a 4 × 4 graphene supercell with the generalized gradient approximation (GGA), specifically the Perdew-Burke-Ernzerhof (PBE) functional; the adsorption energies of −14, −67, and −29 meV were found for CO, NO_2_, and NO molecules, respectively [43]. At the same time, Wehling et al. conducted the first joint experimental and theoretical investigation of the NO_2_ adsorption on graphene. To this end, they used the local density approximation (LDA) and the GGA for their calculations [44]. The computed NO_2_ adsorption energy with the GGA method was similar to that reported by Leenaerts et al. [43]. However, the NO_2_ adsorption energy calculated using the LDA method was higher that the computed energy that employed the GGA method [44]. In another investigation, Lin et al. studied the CO, and NO_2_ adsorption on graphene using a 4 × 4 graphene supercell with the van der Waals density functional (vdW-DF2) and LDA methods [45]. The CO and NO_2_ adsorption energies calculated by vdW-DF2 were larger than those obtained by LDA [45]. For the adsorption mechanism of the toxic gases on the pristine graphene, for the CO and NO molecules, the most stable interaction occurs when the CO [43,45] and NO [43] molecules are parallel to the graphene surface, whereas for the NO_2_ molecule, the most stable interaction is with the O atoms of the N-O bonds pointing toward the graphene surface [43,44,45]. Although the adsorption energies of the gases on graphene are notably affected by the methods employed [44,45,46], the interaction between gases and pristine graphene is weak [43]. This could limit the sensitivity of pristine graphene to detecting toxic gases.

## 3. Pristine Graphene Decorated with Transition Metals

**Figure 3:**
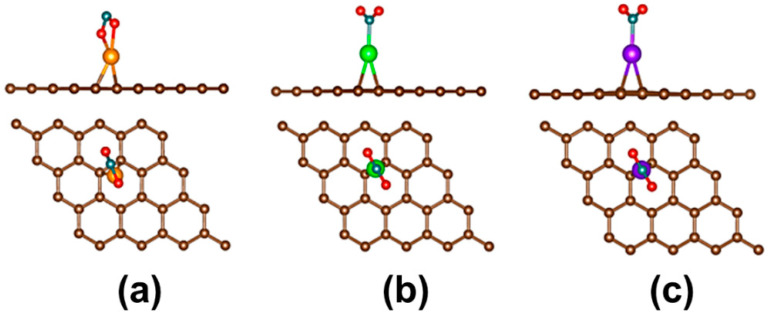
Top view (top) and side view (bottom) of optimized geometry for NO_2_ adsorbed on (**a**) Ni/graphene decorated with Ni, (**b**) graphene decorated with Pd, and (**c**) graphene with Pt. Figure obtained from [51].

To date, researchers have employed different strategies for improving the reactivity of pristine graphene toward the detection of toxic gases. One of these strategies is the use of pristine graphene decorated with transition metals. This strategy involves the deposition of transition metal atoms onto the pristine graphene. To date, various DFT studies on toxic gas adsorption on pristine graphene decorated with transition metals are available in the literature [47,48,49,50,51]. In the first instance, the CO, NO, and SO_2_ adsorption on Co-decorated graphene was studied [47]. Lately, the NO [48] and SO_2_ [49] adsorption on Pt-decorated graphene was investigated. In another study, the CO and NO adsorption on Li-decorated graphene was calculated [50]. Finally, the NO_2_ adsorption on Ni-, Pd-, and Pt-decorated graphene was computed [51]. On the interaction mechanism between the toxic gases and graphene decorated with transition metals; for the CO and NO molecules, the most stable interaction occurs when the CO [47,50] and NO [47,48,50] molecules are vertical to the graphene decorated with transition metals. Furthermore, it has been reported that the atom type used to decorate the graphene can influence on the adsorption mechanism between the toxic gas and the graphene [51]. For instance, the mode of NO_2_ adsorption on the graphene decorated with Ni is different with respect to the graphene decorated with Pd and Pt (see Figure 3).

The adsorption energies of toxic gases on graphene decorated with transition metals is much higher than those on pristine graphene (see Table 2). Such increments in the adsorption energy can be attributed to the modification of the electronic properties of transition metals-decorated graphene compared to undecorated pristine graphene. For example, a high charge transfer from metallic atoms to the graphene has been observed, which improves the reactivity of pristine graphene [48,49]. All previous results demonstrated that the toxic gases adsorption energies were enhanced on graphene decorated with transition metals compared to the adsorption energies on pristine graphene. This shows that pristine graphene decorated with transition metals is a promising material for use in toxic gas sensors. However, to date, DFT studies on the selectivity of graphene decorated with transition metals toward toxic gases have scarcely been reported in literature. Therefore, more theoretical studies on the selectivity of pristine graphene decorated with transition metals should be carried out.

## 4. Defective Graphene

**Figure 4:**
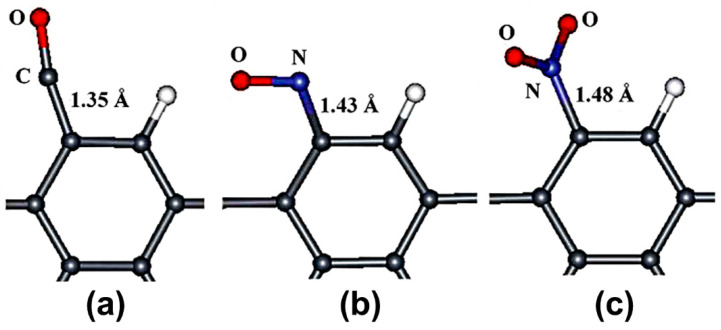
Optimized structures of armchair graphene nanoribbons (AGNRs) with gas molecules: (**a**) CO, (**b**) NO, and (**c**) NO_2_. It only shows the structure around the adsorbed molecule. Gray, white, red, and blue spheres represent C, H, O, and N atoms, respectively. Figure modified from [56].

Another strategy employed to modify the reactivity of pristine graphene is through defects. As has been reported in literature, nanoscale defects bring new functionalities that could be useful for different applications. For instance, structural defects notably modify the mechanical, chemical, and electronic properties of graphene [52]. At the theoretical level, structural defects have become very important to modify the graphene reactivity because these can be introduced into graphene during synthesis by chemical treatment or irradiation [52,53]. To date, there have been various theoretical studies conducted on the use of defective graphene as toxic gas sensor [54,55,56,57,58,59,60,61,62,63,64,65]. For instance, Huang et al. investigated the CO, NO, NO_2_ on armchair graphene nanoribbons (AGNRs) with edge dangling bond defects using PW91 functional (see Figure 4). The CO, NO, and NO_2_ adsorption energies were −1.34, −2.29, and −2.70 eV, respectively. These results indicate that the toxic gas adsorption at AGNR edges is stronger than on graphene surface [56]. To date, different defects have been introduced in the graphene surface to improve its reactivity toward the toxic gases, see Table 3. The single-vacancy and Stone—Wales defects have been used to modify the graphene surface. The single-vacancy has been the defect mostly studied. The single-vacancy defects in graphene have been found to have stronger interactions with toxic gases compared to pristine graphene. This shows that graphene with single-vacancy defects is a promising material for use in toxic gases sensors. The good sensitivity of graphene with single-vacancy defects is attributed to the modified electronic properties compared to those of pristine graphene. The removed C atom produces the three neighboring C atoms having three dangling bonds, which produce localized states at the Fermi level [59,63]. For the adsorption mechanism of the toxic gases on the graphene with a single-vacancy; in the case of CO and NO molecules, the most stable adsorption occurs when the C and N atoms of the CO [57,58,59,60,61] and NO [57,58,60,63] molecules are in the vacancy of graphene, respectively, whereas for the NO_2_ and SO_2_ molecules, the most stable interaction occurs when the NO_2_ [58] and SO_2_ [64] molecules are vertical to the defective graphene with the N and S atoms toward the vacancy of graphene, respectively.

It has also been shown that an extra electric field serves as a good strategy to enhance the reactivity of defective graphene toward the toxic gases, as it positively affects the material′s electronic properties [61]. Recently, the CO adsorption on a graphene sheet with single-vacancy defects under different electric fields was investigated [61]. The calculated adsorption energies of CO on the single-vacancy defective graphene under an applied electric field of −0.016 a.u. was 62.6% higher than without the electric field [61], which shows that an external electric field offers a good way to enhance the reactivity of defective graphene toward the toxic gases.

When toxic gas sensors are exposed to aerobic environments, interference from other gases will cause false alarms [57]. Therefore, it is essential to explore the selectivity of the graphene-based sensors toward the toxic gases. In this direction, Ma et al. demonstrated that CO and O_2_ molecules are chemisorbed on graphene with single-vacancy defects. This limits selectively toward the CO since O_2_ chemisorption would lead to a false alarm [57]. In another study, the selectivity of graphene with a single-vacancy defect toward the various gases was investigated (e.g., H_2_, N_2_, O_2_, CO, CO_2_, H_2_O, H_2_S, and NH_3_). Five gases (H_2_, O_2_, CO, CO_2_, and NH_3_) exhibited chemisorption, whereas the remaining gases showed physisorption (N_2_, H_2_O, and H_2_S). For the H_2_, O_2_, CO_2_, and NH_3_ chemisorption involves the dissociation of the molecules; namely, O_2_ → O + O. It is remarkable that only the CO molecule remains without dissociation. Therefore, the graphene with a single-vacancy would be more selective toward the CO detection. For instance, it is observed that the O_2_ molecule requires about 5.11 eV to get dissociated and then bind to the vacancy. Whereas the CO molecule avoids paying that huge dissociation energy. This fact shows that graphene with a single-vacancy defect has a higher selectivity toward the CO detection [59].

## 5. Doped Graphene

Another approach widely used to modify the reactivity of pristine graphene is through doping. Doping atoms proved to substantially modify the electronic, chemical, and structural properties of pristine graphene [66,67]. At the theoretical level, there are various routes to dope the graphene sheet. A widely used method is to replace a carbon atom with the doping atom in the graphene sheet. Currently, different doped-graphene sheets have been explored as toxic gas sensors, replacing a carbon atom by a dopant atom [55,68,69,70,71,72,73,74,75,76,77,78,79,80,81,82,83,84,85,86,87,88,89,90,91,92,93,94,95,96,97,98,99,100]. Around 30 elements of the periodic table have been explored for use as doping materials, with N being the most studied element dopant due to its similar atomic radii with C (see Table 4). Among the toxic gases reviewed, CO gas is the most investigated due its high toxicity in humans [101]. It is also observed that the GGA (specifically PBE) method and supercell approach are the most widely approaches used for studying doped graphene for use in toxic gas sensors. Interestingly, several studies consider dispersion corrections in the calculations to better describe the interaction between the toxic gases and doped graphene. According to adsorption energies of the toxic gases, in most cases, it is observed that the toxic gases were adsorbed stronger on doped graphene than on pristine graphene. This shows that the doped-graphene sheets are good candidates as toxic gas sensors. The increase in the adsorption energy can be attributed to the modification of the structural and electronic properties of doped graphene compared to pristine graphene. For instance, a high charge transfer from metallic atoms to the graphene has been observed, which improves the reactivity of doped graphene toward the toxic gases [71,85,99]. However, in some cases, the interaction between the toxic gases and the doped-graphene sheet has been reported to be low, as in the case of the interaction of CO on the N-doped graphene. For the adsorption mechanism of the toxic gases on the doped graphene, it has been reported that the atom type used to dope the graphene can influence on the adsorption mechanism between the toxic gas and the graphene [69,71]. Finally, although all calculations are conducted at the DFT level, there are discrepancies between the results reported (e.g., NO_2_ adsorption energies on the N-doped graphene) in Table 4. These can be attributed to various factors, such as the functional and dispersion corrections employed in the calculations, the type and site of gas adsorption on which the adsorption energy was calculated, among others.

An extra electric field could be a good strategy to enhance the reactivity of graphene-based gas sensors [61]. In this sense, the CO adsorption on Al-doped graphene under different electric fields was investigated [83]. The calculated adsorption energies of CO on the Al-doped graphene under an applied electric field of −0.03 a.u. were higher than those without the electric field [83]. This indicates that an external electric field is a good way to enhance the reactivity of doped graphene toward the toxic gases [83,95]. It can also be used for the desorption of toxic gases on the sensor surface, only modifying the direction of the electric field. In this context, the NO and NO_2_ adsorption on Fe-doped graphene under different electric fields (0.01–0.05 a.u.) was investigated [79]. Electric fields above 0.03 a.u. have been found to cause NO and NO_2_ desorption from the surface of Fe-doped graphene [79]. CO desorption from the Al-doped-graphene surface has also been demonstrated under the application of an electric field ≥0.03 a.u. [83]. Therefore, an electric field can be employed to reactivate the doped-graphene toxic gas sensors for repetitious applications.

On the other hand, the selectivity of doped graphene toward the toxic gases has been investigated [77,79]. In this sense, Cortés-Arriagada et al. investigated the selectivity of Fe-doped graphene toward the CO and SO_2_ molecules in O_2_ environments [77]. They computed an O_2_ adsorption energy of −1.68 eV, which is similar or higher than the energy absorption of CO and SO_2_ molecules. This limits selectively toward the CO and SO_2_ molecules in aerobic environments [77]. Another study examined the selectivity of Fe-doped graphene toward the NO and NO_2_ gases in O_2_ environments [79]. Results showed that Fe-doped graphene is selective toward the NO and NO_2_ molecules in O_2_ environments [79].

**Figure 5:**
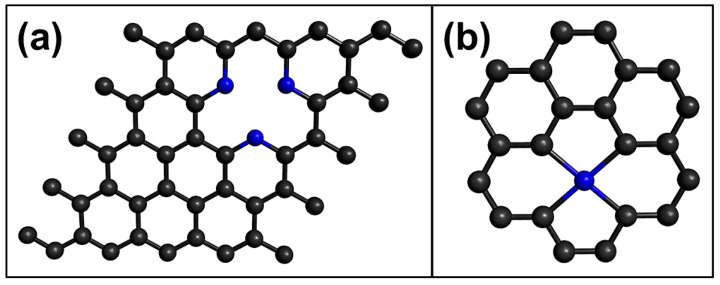
(**a**) Pyridinic-type N-doped graphene, (**b**) doped vacancy-defected graphene. Black and blue spheres represent C and H atoms, respectively.

Another strategy for doping graphene has been to substitute various carbon atoms with the doping atoms. Figure 5a shows a graphene sheet with three atoms of N and a vacancy. This type of doping is known as pyridinic-type doping. Currently, there are some detailed studies on the use of pyridinic-type N-doped graphene (PNG) as toxic gas sensors [57,102]. Ma et al. investigated toxic gas adsorption on PNG sheet using the GGA method [57]. They demonstrated that the PNG is a good candidate for selectively sensing CO from air [57]. Recently, the NO, and SO2 adsorption on PNG was investigated using the B3LYP approximation [102]. It was shown that the NO molecule is weakly adsorbed on PNG sheet [57,102], which shows that PNG may not be a good candidate as a NO sensor. However, the SO_2_ gas is strongly adsorbed (−2.58 eV), thus, PNG may be a good candidate as a SO_2_ sensor.

**Figure 6:**
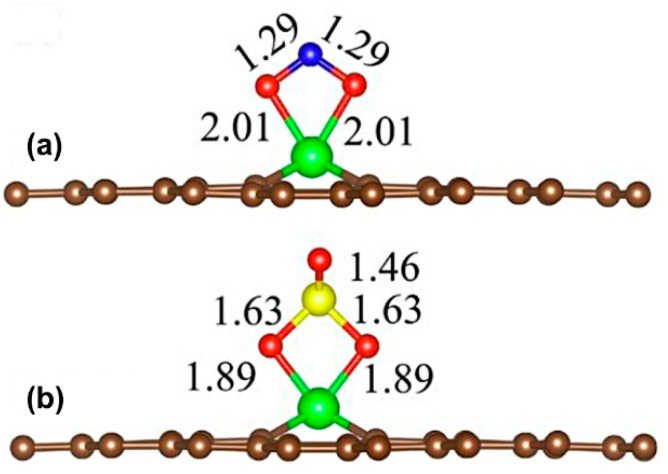
Most stable structures of NO_2_ (**a**) and SO_3_ (**b**) adsorption on Fe-doped vacancy-defected graphene (The bond length in Å). Brown, green, red, blue, and yellow spheres represent C, Fe, O, N, and S atoms, respectively. Figure modified from [80].

Another strategy employed to dope the graphene sheets is inserting the doping atom in a double vacancy (divacancy), see Figure 5b. These structures are interesting because they show better reactivity toward the toxic gases than defective graphene [60]. Consequently, there have been various studies on the use of doped vacancy-defected graphene as toxic gases sensors [60,62,75,80,89]. Jia et al. investigated the CO adsorption on Mn-doped vacancy-defected graphene using the PBE functional [62]. The CO adsorption energy on Mn-doped vacancy-defected graphene was higher than on defective or pristine graphene [62]. In another study, the CO and NO adsorption on Fe-doped vacancy-defected graphene was investigated using the PBE approximation [75]. Adsorption energies of −1.10 and −2.41 eV were computed for CO and NO on Fe-doped vacancy-defected graphene, respectively [75]. At the same time, Gao et al. computed the NO_2_ and SO_3_ interaction on Fe-doped vacancy-defected graphene employing the PBE functional (see Figure 6) [80]. Adsorption energies of NO_2_ (−1.59 eV) and SO_3_ (−1.39 eV) were investigated on Fe-doped vacancy-defected graphene [80]. Recently, Ni-doped vacancy-defected graphene sheets were studied as toxic gases sensors considering the PBE functional [89]. The computed results indicate that NO (−1.87 eV) and NO_2_ (−1.30 eV) were strongly adsorbed on Ni-doped vacancy-defected graphene, while the SO_2_ (−0.36 eV) and SO_3_ (−0.38 eV) gases were weakly adsorbed [89]. Finally, the CO and NO adsorption on Pd-doped vacancy-defected graphene were computed using the PBE functional [60]. The computed adsorption energies of CO and NO molecules on Pd-doped vacancy-defected graphene were higher than on single-vacancy and pristine graphene [60].

Many theoretical studies have been conducted on the use of doped graphene as toxic gas sensors. The results evidence that doped graphene sheets are good candidate materials as gas sensors. To experimentally confirm some of the above-mentioned theoretical predictions, various doped graphene materials have been synthesized and evaluated as toxic gas sensors [103,104,105,106,107,108]. Based on experimental evidence, the sensitivity and selectivity of doped graphene were higher than pristine graphene [103,104,105,106,107]. However, it is difficult to control the doping concentration and the number of graphene layers. Hence, future trends should be focused on the improvement of doped graphene gas sensors through novel, low-cost industrially scalable techniques that allow to control the doping concentration and type in graphene.

## 6. Conclusions and Perspectives

This review presents a detailed and critical analysis of current progress of graphene-based toxic gas sensors using first-principle methods. Following the development of graphene as a gas sensor, it has gained considerable interest from both a theoretical and a technological viewpoint. Therefore, modifications made to graphene to improve the detection of CO, NO_x_, and SO_x_ toxic gases were revised and analyzed in detail. Based on this review, we concluded the following:

(a) The interaction between toxic gases and pristine graphene is weak, which reduces the sensitivity and selectivity of pristine graphene toward the toxic gases.
(b) The pristine graphene decorated with transition metals is a promising material for use in a toxic gas sensor. However, up to now these types of studies are still scarce; therefore, more theoretical studies on the sensitivity and selectivity of pristine graphene decorated with transition metals toward the toxic gases should be carried out.
(c) It was observed that graphene with single-vacancy defects interacts stronger with the toxic gases compared to pristine graphene. Therefore, it is a promising material for use in toxic gas sensors. In addition to point defects, line or multivacancy defects should be investigated at the DFT level, to enrich graphene functionalities.
(d) Bilayer and multilayer graphene exhibit higher different dimensionalities than single-layer graphene, which can increase the number of possible defect types, namely, point defects, line defects, and so on. At the theoretical level, more attention should be paid to understanding stable bilayer and multilayer graphene with randomly distributed defects.
(e) A large number of theoretical studies have addressed the use of doped graphene as a toxic gas sensor. The evidence indicates that doped-graphene sheets are good candidate materials. However, up to date, DFT studies on the selectivity of doped graphene toward the toxic gases are limited. Therefore, more theoretical studies on the selectivity of doped graphene toward the toxic gases should be carried out. In addition, feasible approaches to facilitate the desorption of toxic gas on the doped graphene surface should be investigated.
(f) The pyridinic-type N-doped graphene and doped vacancy-defected graphene are good materials for use in toxic gases sensors. However, more DFT-based studies on pyridinic-type N-doped graphene and doped vacancy-defected graphene as toxic gas sensors are needed.
(g) The reasons for the difference of adsorption energy obtained by using different functionals (e.g., GGA, LDA, PBE, and vdW-DF2) in the calculation methods should be compared and analyzed.
(h) This review shows the importance of theoretical studies for the design of novel and efficient toxic gas sensors. The theoretical results obtained up to now can help and motivate experimental groups to design novel and efficient graphene-based toxic gas sensors.

## Figures and Tables

**Table 1:** Main toxic inhalation hazards.

Gas or Vapor	Irritate	Odor	Signs and Symptoms	Refs.
Carbon monoxide	No	No	Tissue hypoxia, hypoxic cardiac dysfunction, subtle cardiovascular, unconsciousness, and death after prolonged exposures or after acute exposures to high concentrations of CO.	[3,4,5]
Nitrogen oxides	Yes	No	Nausea, headache, respiratory illness (cough and irritation of the respiratory tract), asthma, pneumonia, possibly tuberculosis, and Parkinson’s disease.	[6,7,8,9]
Sulfur oxides	Yes	Yes	Neurological damage, bronchitis, bronchial asthma, emphysema, bronchoconstriction and mucus.	[3,4,10,11]

**Table 2:** Adsorption energies of toxic gases on transition metals decorated graphene.

Material	Gas	E_ads_ (in eV)	Functional	Approach	Refs.
Co-decorated graphene	CO	−2.19	PBE	Supercell	[47]
Co-decorated graphene	NO	−4.04	PBE	Supercell	[47]
Co-decorated graphene	SO_2_	−2.35	PBE	Supercell	[47]
Pt-decorated graphene	NO	−2.06	B3LYP	Supercell	[48]
Pt-decorated graphene	NO_2_	−2.00	PBE	Supercell	[51]
Pt-decorated graphene	SO_2_	−1.58	B3LYP	Supercell	[49]
Li- decorated graphene	CO	−0.55	B3LYP	Finite system	[50]
Li-decorated graphene	NO	−0.14	B3LYP	Finite system	[50]
Ni-decorated graphene	NO_2_	−2.63	PBE	Supercell	[51]
Pd-decorated graphene	NO_2_	−1.59	PBE	Supercell	[51]

**Table 3:** Adsorption energies of toxic gases on defective graphene.

Material	Gas	E_ads_ (in eV)	Functional	Approach	Refs.
Single vacancy	CO	−6.05	GGA	Supercell	[57]
Single vacancy	CO	−2.33	CA-PZ	Supercell	[58]
Single vacancy	CO	−5.06 ^a^, −5.15	PBE	Supercell	[59]
Single vacancy	CO	−0.07	PBE	Supercell	[60]
Single vacancy	CO	−1.86	PBE	Supercell	[61]
Single vacancy	CO	−0.18	PBE	Supercell	[62]
Single vacancy	NO	−6.64	GGA	Supercell	[57]
Single vacancy	NO	−3.04	CA-PZ	Supercell	[58]
Single vacancy	NO	−1.20	PBE	Supercell	[60]
Single vacancy	NO	−8.25	PBE	Supercell	[63]
Single vacancy	NO_2_	−3.04	CA-PZ	Supercell	[58]
Single vacancy	NO_2_	−6.41 ^b^	PBE	Supercell	[63]
Single vacancy	SO_2_	−2.38	PBE	Supercell	[64]
Stone—Wales	CO	−1.30 ^a^		Supercell	[59]
Stone—Wales	SO_2_	−0.19	PBE	Supercell	[65]

^a^ Results obtained using self-consistent-charge density-functional tight-binding method. ^b^ The NO_2_ dissociation into O and NO was observed.

**Table 4:** Adsorption energies of toxic gases on doped graphene.

Material	Gas	E_ads_ (in eV)	Functional	Approach	Refs.
N-doped	CO	−0.14	CA-PZ	Supercell	[58]
N-doped	CO	−0.03	PBE	Supercell	[68]
N-doped	CO	−0.13	PBE	Finite system	[69]
N-doped	CO	−0.01	PBE	Supercell	[70]
N-doped	NO	−0.40	CA-PZ	Supercell	[58]
N-doped	NO	−0.08	PBE	Supercell	[68]
N-doped	NO	−0.09	PBE	Supercell	[70]
N-doped	NO	0.16	PBE	Supercell	[71]
N-doped	NO_2_	−0.98	CA-PZ	Supercell	[58]
N-doped	NO_2_	−0.26	PBE	Supercell	[70]
N-doped	NO_2_	−0.44	PBE	Supercell	[71]
N-doped	SO_2_	−0.29	PBE	Supercell	[68]
N-doped	SO_2_	−0.19	PBE	Supercell	[70]
N-doped	SO_2_	−0.17	PW91	Supercell	[72]
N-doped	SO_2_	−0.29	B3LYP	Finite system	[73]
N-doped	SO_3_	−0.68	B3LYP	Finite system	[73]
Fe-doped	CO	−1.71	PBE	Supercell	[74]
Fe-doped	CO	−1.45	PBE	Supercell	[75]
Fe-doped	CO	−1.46	B3LYP	Finite system	[76]
Fe-doped	CO	−1.60	PBE	Finite system	[77]
Fe-doped	CO	−1.50	B3LYP	Finite system	[78]
Fe-doped	NO	−2.40	PBE	Supercell	[74]
Fe-doped	NO	−2.24	PBE	Supercell	[75]
Fe-doped	NO	−2.23	PBE	Finite system	[79]
Fe-doped	NO_2_	−2.19	PBE	Finite system	[79]
Fe-doped	NO_2_	−2.20	PBE	Supercell	[80]
Fe-doped	SO_2_	−1.68	PBE	Supercell	[74]
Fe-doped	SO_2_	−1.80	PBE	Finite system	[77]
Fe-doped	SO_3_	−1.81	PBE	Supercell	[80]
B-doped	CO	−0.14	CA-PZ	Supercell	[58]
B-doped	CO	−0.13	PBE	Finite system	[69]
B-doped	CO	−0.02	PBE	Supercell	[70]
B-doped	NO	−1.07	CA-PZ	Supercell	[58]
B-doped	NO	−0.34	PBE	Supercell	[70]
B-doped	NO_2_	−1.37	CA-PZ	Supercell	[58]
B-doped	NO_2_	−0.33	PBE	Supercell	[70]
B-doped	SO_2_	−0.03	PBE	Supercell	[70]
B-doped	SO_2_	−0.21	PW91	Supercell	[72]
B-doped	SO_2_	−0.12	B3LYP	Finite system	[81]
B-doped	SO_3_	−0.18	B3LYP	Finite system	[81]
Al-doped	CO	−0.77	PBE	Finite system	[69]
Al-doped	CO	−0.66	PBE	Supercell	[70]
Al-doped	CO	−4.98	PBE	Supercell	[82]
Al-doped	CO	−0.57 ^a^	PBE	Supercell	[83]
Al-doped	CO	−0.56	B3LYP	Supercell	[84]
Al-doped	NO	−1.35	PBE	Supercell	[70]
Al-doped	NO_2_	−2.48	PBE	Supercell	[70]
Al-doped	NO_2_	−0.65	B3LYP	Supercell	[85]
Al-doped	SO_2_	−1.65	PBE	Supercell	[65]
Al-doped	SO_2_	−1.54	PBE	Supercell	[70]
Al-doped	SO_2_	−1.26	PW91	Supercell	[72]
Pd-doped	CO	−0.91	PBE	Supercell	[60]
Pd-doped	CO	−0.92	B3LYP	Finite system	[76]
Pd-doped	CO	−1.05	PBE	Supercell	[86]
Pd-doped	CO	−1.07	PBE	Supercell	[87]
Pd-doped	NO	−3.92	PBE	Supercell	[60]
Pd-doped	NO	−1.33	PBE	Supercell	[82]
Pd-doped	NO_2_	−2.17	PBE	Supercell	[87]
Pd-doped	NO_2_	−2.19	PBE	Supercell	[87]
Pd-doped	SO_2_	−1.12	PBE	Supercell	[87]
Pd-doped	SO_2_	−5.78	PBE	Supercell	[88]
Ni-doped	CO	−1.02	B3LYP	Finite system	[76]
Ni-doped	CO	−0.96	B3LYP	Finite system	[78]
Ni-doped	NO	−1.64	PBE	Supercell	[89]
Ni-doped	NO_2_	−1.83	PBE	Supercell	[89]
Ni-doped	SO_2_	−4.21	PBE	Supercell	[88]
Ni-doped	SO_2_	−0.92	PBE	Supercell	[89]
Ni-doped	SO_3_	−1.59	PBE	Supercell	[89]
Ti-doped	CO	−0.45	PBE	Supercell	[68]
Ti-doped	CO	−1.00	B3LYP	Finite system	[78]
Ti-doped	NO	−1.72	PBE	Supercell	[68]
Ti-doped	NO	−1.44	PBE	Supercell	[71]
Ti-doped	NO_2_	−2.98	PBE	Supercell	[71]
Ti-doped	SO_2_	−3.20	PBE	Supercell	[68]
Mn-doped	CO	−1.50	PBE	Supercell	[62]
Mn-doped	CO	−1.42	B3LYP	Finite system	[78]
Mn-doped	NO	−2.14	PBE	Supercell	[90]
Mn-doped	NO_2_	−2.76	PBE	Supercell	[90]
Mn-doped	SO_2_	−1.73	PW91	Supercell	[72]
Mn-doped	SO_2_	−1.83	PBE	Supercell	[90]
Co-doped	CO	−0.94	B3LYP	Finite system	[76]
Co-doped	CO	−0.94	B3LYP	Finite system	[78]
Co-doped	CO	−0.62	PBE	Supercell	[47]
Co-doped	NO	−1.51	PBE	Supercell	[47]
Co-doped	SO_2_	−1.07	PBE	Supercell	[47]
Pt-doped	CO	−1.30	B3LYP	Finite system	[76]
Pt-doped	NO	−6.22	PBE	Supercell	[91]
Pt-doped	NO_2_	−7.37	PBE	Supercell	[91]
Pt-doped	NO_2_	−2.21	PBE	Supercell	[92]
Pt-doped	SO_2_	−1.02	PW91	Supercell	[72]
Pt-doped	SO_2_	−1.06	PBE	Supercell	[92]
Si-doped	CO	−0.25	PBE	Finite system	[69]
Si-doped	NO	−0.82	PBE	Supercell	[93]
Si-doped	NO_2_	−2.17	PBE	Supercell	[93]
Si-doped	SO_2_	−0.90	PW91	Supercell	[72]
P-doped	CO	−0.07	PBE	Supercell	[94]
P-doped	NO	−0.51	PBE	Supercell	[94]
P-doped	NO_2_	−1.89	PBE	Supercell	[94]
P-doped	SO_2_	−0.32	PBE	Supercell	[94]
S-doped	CO	−0.01	PBE	Supercell	[70]
S-doped	NO	−0.12	PBE	Supercell	[70]
S-doped	NO_2_	−0.83	PBE	Supercell	[70]
S-doped	SO_2_	−0.09	PBE	Supercell	[70]
Ga-doped	CO	−0.67	PBE	Supercell	[95]
Ga-doped	NO	−0.78	PBE	Supercell	[95]
Ga-doped	NO	−0.81	PBE	Supercell	[96]
Ga-doped	NO_2_	−1.93	PBE	Supercell	[95]
Ag-doped	NO	−6.93	PBE	Supercell	[91]
Ag-doped	NO_2_	−7.83	PBE	Supercell	[91]
Ag-doped	SO_2_	−0.97	PW91	Supercell	[72]
Au-doped	NO	−8.47	PBE	Supercell	[91]
Au-doped	NO_2_	−9.34	PBE	Supercell	[91]
Au-doped	SO_2_	−1.28	PW91	Supercell	[72]
Cr-doped	CO	−1.63	B3LYP	Finite system	[78]
Cr-doped	SO_2_	−1.68	PW91	Supercell	[72]
Cr-doped	SO_2_	−1.59 ^b^	PW91	Supercell	[97]
Nb-doped	CO	−0.53	PBE	Supercell	[98]
Nb-doped	SO_2_	−0.32	PBE	Supercell	[98]
Ta-doped	NO_2_	−2.31	PBE	Supercell	[99]
Ta-doped	SO_2_	−1.68	PBE	Supercell	[99]
Li-doped	CO	−3.51	PBE	Supercell	[100]
Sc-doped	CO	−0.35	B3LYP	Finite system	[78]
V-doped	CO	−0.55	B3LYP	Finite system	[78]
Cu-doped	CO	−1.20	B3LYP	Finite system	[78]
Zn-doped	CO	−0.67	B3LYP	Finite system	[78]
Ru-doped	CO	−1.22	B3LYP	Finite system	[76]
Rh-doped	CO	−1.01	B3LYP	Finite system	[76]
In-doped	CO	−0.02	PBE	Supercell	[61]
Sb-doped	CO	−0.01	PBE	Supercell	[61]
Os-doped	CO	−1.80	B3LYP	Finite system	[76]
Ir-doped	CO	−1.57	B3LYP	Finite system	[76]

^a^ Results obtained under electric field = 0.0 a.u. ^b^ Adsorption energy calculated using zigzag graphene nanoribbons.

## Data Availability

Not applicable.
